# Acute Stress and Anxiety in Medical Residents on the Emergency Department Duty

**DOI:** 10.3390/ijerph15030506

**Published:** 2018-03-13

**Authors:** Joaquín M. González-Cabrera, María Fernández-Prada, Concepción Iribar, Rogelio Molina-Ruano, María Salinero-Bachiller, José M. Peinado

**Affiliations:** 1 Faculty of Education, Universidad Internacional de la Rioja, 26006 Logroño, Spain; joaquín.gonzalez@unir.net; 2 Department of Preventive Medicine, Central Hospital of Asturias, 33006 Oviedo, Spain; mariafdezprada@gmail.com; 3 Department of Biochemistry, Molecular Biology and Immunology 3, University of Granada, 18016 Granada, Spain; mciribar@ugr.es (C.I.); msaliner@correo.ugr.es (M.S.-B.); 4 Institute of Neuroscience “Federico Olóriz”, University of Granada, 18012 Granada, Spain; 5 Emergency Department, Virgen de las Nieves University Hospital, 18014 Granada, Spain; rogeliomolina22@gmail.com

**Keywords:** acute stress, cortisol, medical resident, Emergency Department-duty day, anxiety

## Abstract

The objectives of this longitudinal study were to compare salivary cortisol release patterns in medical residents and their self-perceived anxiety levels between a regular working day and a day when on call in the emergency department (ED-duty day) and to determine any differences in cortisol release pattern as a function of years of residency or sex. The study included 35 residents (physicians-in-training) of the Granada University Hospital, Granada, Spain. Acute stress was measured on a regular working day and an ED-duty day, evaluating anxiety-state with the Spanish version of the State-Trait Anxiety Inventory. Physiological stress assessment was based on salivary cortisol levels. Cortisol release concentrations were higher on an ED-duty day than on a regular working day, with a significantly increased area under the curve (AUC) (*p* < 0.006). This difference slightly attenuated with longer residency experience. No gender difference in anxiety levels was observed (*p* < 0.001). According to these findings, the hypothalamic-pituitary-adrenal axis activity and anxiety levels of medical residents are higher on an ED-duty day than on a regular working day.

## 1. Introduction

It should be kept in mind that stress can be positive (eustress) or negative (distress). Thus, eustress is essential to grow, develop, and achieve high levels of performance in a wide range of tasks and activities. In contrast, distress is considered a potential source of physical and psychological problems, especially when chronic [1,2]. Anxiety and stress are widespread psychological disorders. In a European study, 13.6% of participants reported an anxiety episode at some point in their life and 6.4% described this experience during the previous year [3]. Acute and chronic stress has been associated with physiological impairments [4,5], psycho-somatic diseases and, especially, cardiovascular disease [6]. It has been reported that anxiety levels are higher in healthcare professionals than in the general population, attributed to frequent night-time working, with few hours of sleep, and exhausting workloads, among other causes [7]. This situation is especially relevant in the case of younger physicians with limited experience, who work in an environment of uncertainty [8,9]. Activation of the hypothalamic-pituitary-adrenocortical axis (HPA) by stress is associated with salivary cortisol release, which is increasingly used as a hormonal biomarker for stress [10,11]. Cortisol release follows a daily circadian rhythm, being elevated after awakening and relatively reduced in the evening [12]. Cortisol release can be quantified by constructing the area under the curve (AUC). The typical increase in cortisol levels at 20–30 min after awakening in the morning is known as the “cortisol awake response” (CAR) [13]. Variations in cortisol release have also been associated with age, sex, genetic, and environmental factors [14], and release can be increased by pain, hypoglycemia, exercise, and surgery, among others [15]. A meta-analysis of 208 studies on acute stressors in laboratory settings [16] reported a maximum cortisol response at 20–40 min after a stressful event, while stress research in real-life settings also revealed significant changes in CAR [17]. In clinical studies, it has been reported that cortisol levels are higher with greater severity of illness [18,19]. In addition, a real-life longitudinal study revealed impaired patterns of cortisol release in medical students when facing examinations for a position as resident [20] or when delivering a presentation in public [21]. During their training period (residency), especially when on emergency department (ED) duty, young physicians are given a progressively increasing workload paralleled by an increasing level of related stress [22,23]. Together with sleep deprivation and long work hours, their elevated stress might compromise the correct management of patients [24], and one study reported that relevant levels of anxiety can be experienced by 20–30% of medical residents [9]. Our study hypotheses were that higher anxiety-state levels in residents on days that included an on-call shift in the emergency department (ED-duty day) would impact on their normal pattern of cortisol release and that these effects would be attenuated as residents became more experienced. The objectives of the study were to compare the patterns of release of salivary cortisol and perceived anxiety-state levels in medical residents between a regular working day and an ED-duty day, and to compare cortisol release patterns as a function of years of residency.

## 2. Methods and Subjects

### 2.1. Population and Study Sample

A longitudinal study was conducted during February and March 2015. The eligible population comprised 88 medical residents: 41 first-year (R1), 34 second-year (R2), and 13 third-year (R3) residents. After application of inclusion and exclusion criteria, 40 residents were enrolled and signed their approval for participation was obtained from 40 of the eligible residents, but two of these dropped out and three were lost due to the incorrect collection of saliva samples. Therefore, the final study sample comprised 35 residents (19 R1, 11 R2, and 5 R3), 11 males (31%) and 24 females (69%), with a mean (±standard deviation, SD) age of 26.39 (1.56) years (range, 25–30 years). Participants reported a mean of 6.90 (0.57) h of sleep and a mean of 68.33 (18.25) h on ED duty/month, ranging from 40 to 140 h/month. In our setting, the standard shift for residents is from 8:00 to 15:00 h, and this is extended until 08:00 h on the next day for the ED duty shift, which is compulsory for residents of all specialties. The study inclusion criterion was to have been on duty for at least 40 h/month in the ED of our hospital. Exclusion criteria were: (a) a reported traumatic psychological event in the previous six months; (b) pregnancy; (c) failure to collect saliva correctly; (d) use of medication that might interfere with cortisol metabolism and release (e.g., oral contraceptives); and (e) performance of vigorous physical exercise >2 h/day.

### 2.2. Data Collection

(1) Data were gathered on sex, age, hours of sleep on study days, mean number of hours on ED duty/month, and year of residency (to analyze possible interferences with cortisol release patterns) [25].
(2) Saliva samples were collected in Salivette tubes (Sarstedt International, Nümbrecht, Germany), centrifuged at 2555× *g*. for 8 min and stored at −22 °C until further analysis. Salivary cortisol concentrations (µg/dL) were measured using an electrochemiluminescence immunoassay (Elecsys Cortisol test kit, Roche Diagnostics, Barcelona, Spain) on a Cobas c8000 analyzer (Roche Diagnostics). Salivary cortisol is a valid and reliable marker of the level of this hormone in plasma [16] and its collection is non-invasive, with minimal impact on daily life [25].
(3) State-Trait Anxiety Inventory (STAI) [26]. This stress assessment instrument measures two dimensions of anxiety: trait anxiety (T) and state anxiety (S). We used the state anxiety subscale (STAI-S), which has adequate validity and reliability and has been validated for Spanish populations [27].

### 2.3. Procedure

Informed written consent was obtained from all participants. Each participant collected saliva samples on a regular working day and then, within a maximum of 20 days, on an ED-duty day. On each collection day, a sample of saliva was collected at six time points: (1) immediately after awakening (±07:00 h); (2) 30 min after awakening; (3) 11:00 h; (4) 15:00 h (at end of workday or beginning of duty shift); (5) 20:00 h; and (6) 23:00 h [28]. Psychological stress variables were also assessed (between 08:00 h and 09:00 h) on both a regular working day and an ED-duty day.

### 2.4. Ethical Considerations

Participation in the study was voluntary and anonymous. Participants received no compensation and were informed that they could withdraw at any time without disadvantage. The study was approved by the Biomedical Research Ethics Committee of Granada Hospital, Spain.

### 2.5. Data Analysis

SPSS 20.0 software (IBM, Armonk, NY, USA) was used for statistical analyses, and graphs were plotted with SigmaPlot 11.0 (Systat Software, San Jose, CA, USA). Because the data were non-normally distributed (Shapiro-Wilk test), they were first logarithmically transformed (ln(x + 1)) and then analyzed with Levene’s test to confirm the variance homogeneity. Descriptive statistical analysis (measures of central tendency/dispersion and frequencies) was followed by application of the bilateral Student’s *t*-test for dependent (related) samples, the chi-square (χ^2^) test, and one-factor ANOVA, and construction of the AUC using the trapezoidal method in hours [29]. Cohen’s *d* was determined as a measure of the effect size. CAR values were calculated by subtracting cortisol levels at awakening from cortisol levels 30 min later [28]. *p* < 0.05 was considered significant in all tests.

## 3. Results

### 3.1. Comparison of Salivary Cortisol between Regular Working Days and ED-Duty Days

Table 1 compares cortisol concentrations at each time point between regular working days and ED-duty days.

**Figure 1:**
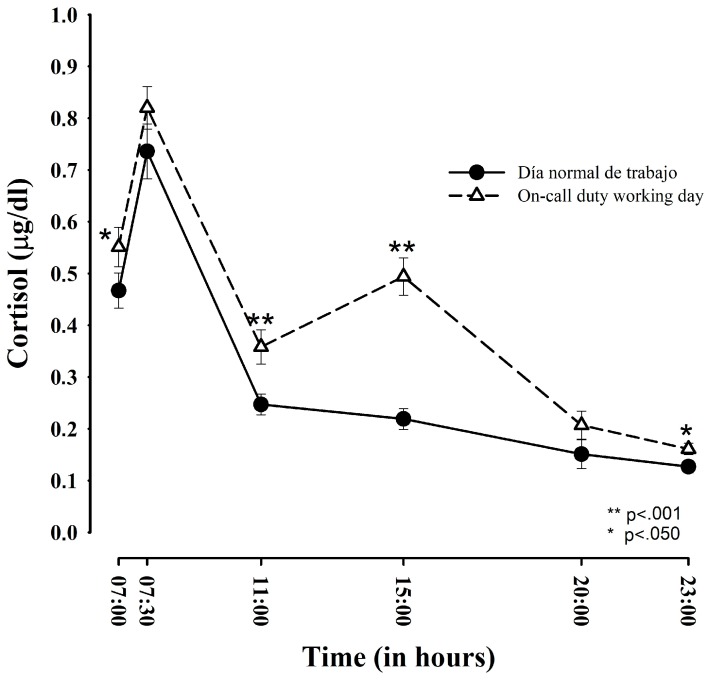
Comparison of salivary cortisol release profiles between regular working day and emergency department (ED)-duty day (*n* = 35).

Figure 1 displays the standard circadian release pattern of participants on all studied days, with an increase upon awakening and then a progressive decrease, reaching the lowest values before going to bed. Salivary cortisol levels were significantly higher on ED-duty days than on regular working days at 7:00 h. (*p* < 0.05), 11:00 h. (*p* < 0.001), 15:00 h. (*p* < 0.001), and 23:00 h. (*p* < 0.05). The significantly higher cortisol release peak at 15:00 h on ED-duty days coincides with the start of the duty shift.

Table 2 exhibits the AUC and CAR results obtained. ED-duty days and regular working days only significantly differed in AUC values (*p* < 0.001).

### 3.2. Socio-Demographic Variables and Salivary Cortisol Release

**Figure 2:**
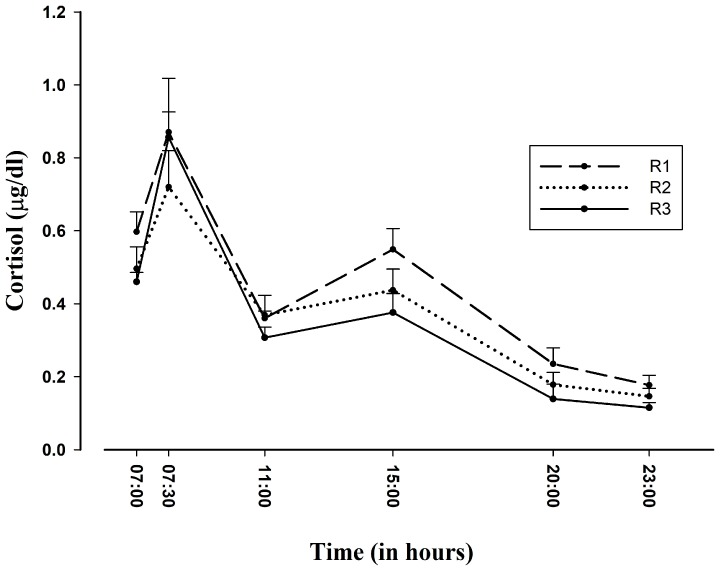
Cortisol profile on ED-duty days as a function of year of residency (arithmetic mean (± SEM) in 35 residents). SEM: standard error of the mean; R1: first year of residency; R2: second year of residency; R3: third year of residency.

No significant gender differences were found in cortisol release, AUC, or CAR values on either regular working days or ED-duty days (χ^2^ = 32.550, *p* < 0.296). Likewise, no significant differences were observed in cortisol release levels by year of residency (F_2,32_ = 1.683, *p* < 0.202) (see Figure 2), age (F_5,29_ = 0.337, *p* < 0.886), hours of sleep (F_2,32_ = 2.626, *p* < 0.088), or number of hours on duty (F_10,24_ = 2.414, *p* < 0.057) on either ED-duty days or regular working days.

### 3.3. Psychological Scores in Anxiety-State

STAI-S results were compared with reference population values published by Spielberger et al. [26] and Guillén-Riquelme and Buela-Casal [27]. In comparison with the former, a significant increase in state anxiety was only observed in female residents on ED-duty days. In comparison with the latter reference values (Guillén-Riquelme and Buela-Casal), a significant increase in anxiety was recorded in both male and female residents on ED-duty days (see Table 3).

STAI-state results also showed that anxiety levels were significantly higher on ED-duty days than on regular working days in both males (*p* < 0.001) and females (*p* < 0.001).

## 4. Discussion

According to the results of this study of medical residents in a real-life setting, salivary cortisol levels and anxiety are increased on ED-duty days. Controversial findings have been published on cortisol response to stress in healthcare professionals, with some authors reporting higher cortisol concentrations than described for the general population among emergency physicians [19] and surgeons [30], while others have found no differences among emergency physicians [31] or intensive care unit nurses [32]. In the present study, cortisol levels of medical residents were increased on ED-duty days but not on regular working days. In comparison to a regular working day, the increase in cortisol release was statistically significantly higher upon awakening (*p* < 0.05) and was even more significant at 11:00 h, four hours before starting the ED duty shift (*p* < 0.01), and at 15:00 h, when this shift started (*p* < 0.01). These significant increases suggest a physiological response to anticipatory stress [20,33] that may possibly result from the emotional “footprint” left by previous experience on ED shifts. A similar anticipatory response was observed before official examinations for medical specialty training (Médico Interno Residente, MIR) positions [20,33]. These changes in the cortisol release pattern were verified by the AUC curve results, which showed significantly larger values on ED-duty days, and this appears to be associated with specific time periods of negative mood or self-perceived stress rather than with chronic stress [34]. In fact, both on regular working days and ED-duty days, cortisol release followed the standard pattern, with the highest values at 30 min after awakening and the lowest at the end of the day.

CAR values did not significantly differ between ED-duty days and regular working days and have been described as more precise than AUC for the evaluation of chronic stress [17,35], although this has not been conclusively established [13]. In the present study, the AUC was significantly larger on ED-duty days than on regular working days; however, between-day differences in CAR values did not reach significance, suggesting that the stress caused by night duties is acute.

Unexpectedly, no relationship was observed between cortisol levels and year of residency. Hence, our hypothesis of an attenuation in cortisol-measured stress with greater experience was rejected, although the peak of cortisol at the start of duty (15:00) tended to flatten with more years of residency. This contrasts with findings in teachers of an improvement in HPA adaptation with longer experience [36]. These data point to a high alteration of HPA that is not significantly reduced by experience, although we observed a progressive reduction between the first and third year of residence. In terms of the eustress-distress continuum, the alteration in the first-year residents may have been more related to distress, due especially to their scant clinical experience, whereas eustress might be more likely to play a role in the HPA alteration observed in the second- and especially third-year residents. However, further research is required to elucidate this issue.

Psychological stress (anxiety) scores paralleled the increase in cortisol release on ED-duty days, when the anxiety scores for both male and female residents were significantly higher than for the reference population published by Guillén-Riquelme and Buela-Casal [27] and those for the females were higher than for the population described by Spielberg [26]. These data are in line with the findings of Buddeberg-Fischer et al. [9].

No significant relationship was observed between cortisol levels and age, hours on duty per month, or hours of sleep, which are potential confounders [25]. Most stress induction studies in laboratory settings have reported higher cortisol levels in males than in females [16,25], whereas real-life investigations have found higher levels in females [31,37] or, as in the present study, no difference between sexes [11,19,20].

Study limitations include the small sample size, although it is similar to some previous investigations [15,19,20,38,39,40], and the failure to control for other confounders (e.g., menstrual cycle, medical specialty, etc.). The reduction from 88 to 35 participants largely resulted from the application of study eligibility criteria but may have caused some selection bias. A further limitation is associated with the use of non-equivalent control groups to compare among different years of residence. Longitudinal studies with a repeated-measures design are warranted to follow residents from the beginning to end of their specialty training and to take into account additional psychological constructs such as depression, which has been associated with cortisol changes.

## 5. Conclusions

This study contributes to the scant literature on patterns of cortisol release in healthcare professionals and provides complementary psychological data [12,13,28,29]. These findings indicate an HPA alteration on ED-duty days, especially during the first years of residency, suggesting the need for emotional and stress control interventions to reduce the stress of residents on ED-duty days and its negative impact on their quality of life and health [41] as well as its potentially negative effects on patient safety.

## Figures and Tables

**Table 1:** Mean cortisol values (*n* = 35 residents) on normal versus emergency department (ED)-duty days at each collection time.

Time points	Regular Work Day	ED-Duty Day	Student’s *t*-Test (t)	*p* Value	Effect Size (*d*)
Upon awakening	0.467 (± 0.198) ^a^ 0.375 (± 0.127) ^b^	0.551 (± 0.221) 0.429 (± 0.136)	2.033; (−0.108; −0.001)	0.050 *	0.41
After 30 min	0.736 (± 0.310) ^a^ 0.538 (± 0.167) ^b^	0.819 (± 0.279) 0.587 (± 0.154)	−1.635; (−0.109; 0.012)	0.112	0.30
At 11:00 h	0.247 (± 0.116) ^a^ 0.217 (± 0.087) ^b^	0.358 (± 0.192) 0.298 (± 0.126)	−5.686; (−0.109; −0.051)	0.001 *	0.75
At 15:00 h	0.219 (± 0.119) ^a^ 0.193 (± 0.092) ^b^	0.494 (± 0.210) 0.392 (± 0.139)	−8.283; (−0.248; −0.151)	0.001 *	1.68
At 20:00 h	0.151 (± 0.109) ^a^ 0.137 (± 0.086) ^b^	0.207 (± 0.157) 0.181 (± 0.110)	−1.966; (−0.090; 0.001)	0.058	0.44
At 23:00 h	0.127 (± 0.074) ^a^ 0.117 (± 0.064) ^b^	0.161 (± 0.102) 0.146 (± 0.081)	−2.040; (−0.057; −0.001)	0.050 *	0.40

^a^ real values expressed in μg/dL; ^b^ logarithmic transformations ln(x + 1); * statistically significant difference.

**Table 2:** Mean area under the curve (AUC) and cortisol awake response (CAR) values for the 35 residents on regular working day versus ED-duty day.

	Total AUC	Bilateral Student’s *t* and Cohen’s *d*	CAR	Bilateral Student’s *t* and Cohen’s *d*
Regular working day	8.777 (± 3.811) ^a^ 2.214 (± 0.362) ^b^	*t* = −7.959 *p* < 0.001 * (−0.513; −0.304) *d* = 1.15	0.2689 (± 0.238) 0.2213 (± 0.285)	*t* = −0.064 *p* < 0.950 (0.044; −0.093) *d* = 0.01
Day on duty	13.620 (± 5.199) ^a^ 2.623 (± 0.352) ^b^		0.2682 (± 0.214) 0.2242 (± 0.165)	

^a^ real values in μg/dL; ^b^ logarithmic transformations ln(x + 1); * statistically significant difference; AUC: area under the curve; CAR= cortisol awake response

**Table 3:** State-Trait Anxiety Inventory (STAI)-State anxiety scores on regular working day versus ED-duty day and comparison with population reference values of Spielberger et al. [26] and Guillén-Riquelme and Buela-Casal [27].

	Regular Working Day		ED-Duty Day	
	Males	Females	Males	Females
	*n* = 11	*n* = 24	*n* = 11	*n* = 24
STAI-State (comparison with data from Spielberger et al. [26])	19.08 (±5.07)	20.17 (±5.24)	23.42 (±5.93)	25.70 (±5.82)
	*p* = 0.344	*p* = 0.009 *	*p* = 0.121	*p* = 0.037 *
	*t* = −0.988	*t* = −2.858	*t* = 1.680	*t* = 2.217
STAI-State (comparison with data from Guillén-Riquelme and Buela-Casal [27])	19.08 (±5.07)	20.17 (±5.24)	23.42 (±5.93)	25.70 (±5.82)
	*p* = 0.052	*p* = 0.085	*p* = 0.001 *	*p* = 0.001 *
	*t* = 2.180	*t* = 1.805	*t* = 4.408	*t* = 6.936

Reference population state anxiety subscale (STAI-S) values are 20.54 for males and 23.30 for females according to Spielberger et al. [26], and 15.87 for males and 18.20 for females according to Guillén-Riquelme and Buela-Casal [27]; * statistically significant difference.
